# Changes in weight secondary to improved odor perception in chronic rhinosinusitis with nasal polyps’ patients treated with Dupilumab

**DOI:** 10.1007/s00405-024-09021-0

**Published:** 2024-10-15

**Authors:** Cecilia Rosso, Eugenio De Corso, Anastasia Urbanelli, Gianluca Fadda, Alberto Maria Saibene, Francesco Ferella, Camilla Spanu, Carlotta Pipolo

**Affiliations:** 1 Otorhinolaryngology Unit, ASST Santi Paolo e Carlo, Università degli Studi di Milano, Milan, Italy; 2 Otorhinolaryngology Unit, A. Gemelli University Hospital Foundation IRCCS, Rome, Italy; 3 Otorhinolaryngology Unit, Department of Surgical Sciences, University of Turin, Turin, Italy; 4 Department of Otolaryngology, San Luigi Gonzaga Hospital, University of Turin, Orbassano, 10043 Italy; 5 Department of Health Sciences, Università degli Studi di Milano, Milan, Italy; 6 Department of Head, Neck and Sensory Organs, Catholic University of The Sacred Heart, Rome, Italy; 7 via Antonio di Rudinì 8, ASST Santi Polo e Carlo, Milan, Italy

**Keywords:** Chronic rhinosinusitis with nasal polyps, Biologics, BMI, Dupilumab, Olfaction, Hyposmia

## Abstract

**Purpose:**

The advent of biologic therapies, notably Dupilumab, has transformed therapeutic approaches to nasal polyposis. This retrospective multicentric study aimed to investigate weight changes in CRSwNP patients undergoing Dupilumab treatment and explore potential correlations with olfactory improvement.

**Methods:**

Ninety-six patients with CRSwNP were followed for at least 12 months, with assessments including BMI (Body Mass Index), olfactory function, and disease severity.

**Results:**

Significant increases in BMI and olfactory perception were observed after 1 year of Dupilumab treatment (*p* < .001). Subgroup analysis showed that patients with hyposmia and normosmia at T12 (1-year follow up) experienced significant weight gain (*p* < .001) alongside improved olfaction (both *p* < .001). Conversely, patients with anosmia after 1 year of therapy and also patients with stable or worsened olfaction did not show significant BMI changes (respectively *p* = .201 and *p* = .107).

**Conclusion:**

While these findings suggest a correlation between olfactory improvement and weight gain/BMI, factors like improved nasal airflow and corticosteroid cessation under Dupilumab treatment may also influence weight in CRPwNP patients. The study highlights the need for further research to elucidate the causal relationship and long-term implications of Dupilumab-induced olfactory improvement on weight regulation.

## Introduction

Chronic rhinosinusitis with nasal polyps (CRSwNP) is a complex disorder that directly affects the quality of life, with may associated symptoms [1–5]. Among them, the loss of smell gained importance and hence has been included as a criterion for patient selection and response to therapy in both international expert consensus statements regarding biologics (EUFOREA and EPOS) [1, 2, 6], as well as in their latest joint update of 2023 [7].

Even though olfactory restoration has been described for all three currently available monoclonal antibodies targeting CRSwNP (Omalizumab, Mepolizumab, and Dupilumab), bith indirect meta-analyses and real-life studies demonstrated that Dupilumab may offer a more significant effect on several outcomes, including restitution of the olfactory sense [8–15], especially showing that the restoration of olfactory function resulted as one of the earliest achieved outcomes, often occurring after only one month of treatment [3, 14].

Of further interest are the secondary effects that the restoration of olfaction may have on the CRSwNP patient [16]. Even though we have long known that anosmia does indeed influence dietary intake behavior in humans, it is still unclear if impacting the olfactory system directly affects eating behavior and the energy balance in humans [17, 18]. Even more so, data regarding possible correlations between the improvement of the sense of smell with Dupilumab and potential weight gain/loss during treatment are missing. Only a few papers are available about the link between Body Mass Index (BMI) and Dupilumab, but they focus more on how weight may influence the response to Dupilumab treatment in CRSwNP [19].

This study, therefore, aims to investigate weight changes in patients undergoing treatment with Dupilumab and to determine a potential correlation, while assessing confounders, between the improvement of smell and, if present, the variations of body weight. The primary endpoint of this study was to assess weight variations and, consequently, BMI changes in each subject.

## Materials and methods

This is a retrospective multicentric real-life study involving three 2nd level rhinologic clinics from March 2021 to September 2023. The research was conducted in accordance with Good Clinical Practice and with the requirements of the World Medical Association’s Declaration of Helsinki. We included 96 patients affected by CRSwNP who met the criteria of severe uncontrolled disease according to the Consensus of the Joint Committee of the Italian Society of Otorhinolaryngology:

- Age ≥ 18 years;
- Treatment with Dupilumab in a real-life setting for severe uncontrolled CRSwNP with a follow-up of at least 12 months (criteria for prescription as found in EPOS, EUFOREA, and the Italian Medicines Agency (AIFA));
- Patients receiving a self-administered subcutaneous 300 mg dose of Dupilumab every 2 weeks and that did not modify the interval dose of administration during the follow-up time.

Patients excluded from the study were those not eligible for Dupilumab treatment, or under other concurrent biological therapies, or with diseases which may alter patient’s metabolism (type-2 diabetes, thyroid dysfunction, etc.). Data about age, sex, comorbidities, previous surgery, and BMI at baseline were collected.

After baseline assessment, follow-up controls were carried out at 14 days, and 1, 3, 6, 9, and 12 months after the treatment start, and included self-assessment questionnaires (SNOT-22, Visual Analogue Scale), body weight, nasal endoscopy to evaluate Nasal Polyp Score (NPS), and Sniffin’ sticks identification test to evaluate the olfactory performance.

The Sniffin’ sticks identification test consists of a clinical sensory test based on 16 pen-like odor-dispensing devices employed to assess the patient’s odor identification using four-alternative forced-choices. The maximum score of the test is 16 points and reflects optimal olfactory function. We assessed anosmia as a Sniffin’ sticks identification test score of 5 or less.

Patients were classified according to the class of smell at baseline and at the end of the study as follows [20]:

- Anosmic group: score of 0–5/16 on the Sniffin’ sticks test.
- Hyposmic group: score of 6–11/16 on the Sniffin’ sticks test.
- Normosmic group: score of 12–16/16 on the Sniffin’ sticks test.

Patients were also classified according to the improvement of olfaction from baseline as follows:

- Olfactory-stable group: patients whose olfaction decreases, stays stable, or does not improve by more than 2 points on the Sniffin’ sticks test evaluation from baseline to T12.
- Smell improvement: improvement of more than 2 points on the Sniffin’ sticks test evaluation from baseline to T12.

During biological therapy, patients underwent continuous treatment with nasal lavages and intranasal corticosteroids (INCS) twice a day. All weight assessments were conducted as per our standard at baseline (start of the treatment with Dupilumab) and subsequently at 3, 6, 9, and 12 months after the beginning of the therapy. Data regarding BMI variation were compared to the scores of the Sniffin’ sticks test at the same time point for each patient.

Statistical analysis was performed using IBM SPSS Statistics software, version 28.0.1.0. Using the Kolmogorov-Smirnoff test, we assessed that BMI data were normally distributed, while Sniffin’ sticks, NPS score, and SNOT-22 score results followed a non-normal distribution. We evaluated the modification of BMI data from the beginning of treatment to a 1-year follow-up using a Paired T-test. The Wilcoxon signed-rank test was then used to evaluate variation of Sniffin’ sticks results from baseline to the first year. We also evaluated whether subgroups (patients with anosmia, hyposmia, normosmia, stability of olfactory function, significant increase in olfactory function) had statistically significant changes in BMI values using the Wilcoxon test, except for the BMI variables of the hyposmic group and smell improvement group, which presented a normal distribution.

## Results

Based on inclusion and exclusion criteria, 96 consecutive patients were enrolled. Demographic and anamnestic data are summarized in Table 1. All patients started with a normal BMI or were overweight at baseline, and none of them encountered so much gain as to change their weight status.

**Table 1:** Demographic and anamnestic data collected at T0 and T12 timepoints

*N*° of patients	96
Age	55.6 (± 12.5)
*Sex*	
males	53 (55.2%)
females	43 (44.8%)
Smokers (n° of patients)	7 (7.3%)
Allergic to inhalants (n° of patients)	42 (43.8%)
Asthmatic patients^1^ (n° of patients)	58 (60.4%)
Non-controlled^2^ asthma (during Dupilumab treatment) (n° of patients)	5 (5.2%)
Non-controlled^3^ CRSwNP (during Dupilumab treatment) (n° of patients)	2 (2.1%)
Previous surgery (n° of patients)	89 (92.7%)
Number of previous surgeries	2.4 (± 1.7)
Months since last surgery	83.6 (± 72.3)
*Quality of smell at baseline*	
Anosmic (0–5/16 at Sniffin sticks)	69 (71.9%)
Hyposmic (6–11/16 at Sniffin sticks)	21 (21.85%)
Normosmic (12–16/16 at Sniffin sticks)	6 (6.25%)
*Quality of smell at T12*	
Anosmic (0–5/16 at Sniffin sticks)	13 (13.5%)
Hyposmic (6–11/16 at Sniffin sticks)	39 (40.62%)
Normosmic (12–16/16 at Sniffin sticks)	44 (45.83%)
Use of OCS during biological therapy (n° of patients)	0

^1^ Asthma intended as moderate or severe asthma [23] ^2^ Non-controlled Asthma defined following International ERS/ATS guidelines [23] ^3^ Non-controlled CRSwNP defined following EPOS guidelines^2^

Statistical analysis revealed a significant increase in the BMI score in patients under Dupilumab treatment at the 1-year follow-up (p = .002; Table 2). Sniffin’ sticks also registered an increase in odor perception at the T12 time point evaluation (*p* < .001, Table 2). NPS and SNOT-22 observed an overall improvement from baseline to the T12 evaluation (Table 2). Univariate regression showing the association between the 2 variables (variation of BMI T0-T12 and Sniffin’ sticks at T12) shows a non-statistically significant association between the two (Spearman correlation; *p* = .181, Table 2).

**Table 2:** Descriptive analysis and statistical comparison of variables

	BMI T0	BMI T12		Sniffin sticks T0	Sniffin sticks T12
*Mean*	25.52	25.93	*Median*	3	11
*SD*	3.83	3.83	IQR	4	5
	BMI T12-T0			Sniffin sticks T12-T0	
*Comparison (p)* ^*a*^	0.002		*Comparison (p)* ^*b*^	< 0.001	
	SNOT22 T0	SNOT22 T12		NPS T0	NPS T12
*Median*	59.50	15.50	*Median*	6	1
*IQR*	20.25	14.75	IQR	2	3
	SNOT22 T12-T0			NPS T12-T0	
*Comparison (p)* ^*b*^	< 0.001		*Comparison (p)* ^*b*^	< 0.001	
	BMI – Sniffin Sticks T12-T0				
*Comparison (p)* ^*c*^	0.181				

^a^ Analysis was made using Paired T-Test since the gaussian distribution of the groups; ^b^ Analysis was made using Wilcoxon signed-rank Test since the non-gaussian distribution of the variables. ^c^ Analysis of the association between the two variables (BMI and Sniffin sticks test) was made using Spearman correlation test

Among patients who had no recovery of olfaction and remained anosmic through T1-T12 (n = 13) (persistence of score of 0–5/16 at T12 evaluation), there was no statistically significant change in BMI values (Wilcoxon test, p = .201). Accordingly, patients whose olfaction decreased, stayed stable, or did not improve by more than 2 points on the Sniffin’ sticks test did not have a significant increase in their BMI score (*n* = 24) (*p* = .107). However, hyposmic patients (6–11/16 on the Sniffin’ sticks test at T12 evaluation) had a statistically significant increase in BMI (*p* = .007) and also an improvement in the Sniffin’ sticks test score (*p* < .001).

Patients who, in general, had a satisfactory improvement in smell (n = 72) (intended as an improvement of more than 2 points on the Sniffin’ sticks score from T0 to T12 evaluation), regardless of their smell class at T12, presented a statistically significant increase in the BMI index (*p* < .001, see Table 3).

**Table 3:** Descriptive analysis and statistical comparison of variables among anosmic, hyposmic, olfactory-stable and smell improvement groups of patients

**Anosmic group** ^a^ **(** ***n*** ** = 13)**					
	BMI T0	BMI T12		Sniffin sticks T0	Sniffin sticks T12
*Median*	26.50	27.70	*Median*	3	3
*IQR*	2.42	3.4	IQR	2.5	2.5
	BMI T12-T0			Sniffin sticks T12-T0	
*Comparison (p)* ^*e*^	0.201		*Comparison (p)* ^*e*^	0.627	
**Hyposmic group** ^b^ **(** ***n*** ** = 39)**					
	BMI T0	BMI T12		Sniffin sticks T0	Sniffin sticks T12
*Median*	25.00	25.10	*Median*	3	10
*IQR*	5.24	4.35	IQR	3	3
	BMI T12-T0			Sniffin sticks T12-T0	
*Comparison (p)* ^*f*^	< 0.001		*Comparison (p)* ^*e*^	< 0.001	
**Normosmic group** ^**b**^ **(** ***n*** ** = 44)**					
	BMI T0	BMI T12		Sniffin sticks T0	Sniffin sticks T12
*Median*	24.60	25.10	*Median*	5	13
*IQR*	5.77	5.96	IQR	7	2
	BMI T12-T0			Sniffin sticks T12-T0	
*Comparison (p)* ^*e*^	< 0.001		*Comparison (p)* ^*e*^	< 0.001	
**Olfactory-stable group** ^**c**^ **(** ***n*** ** = 24)**					
	BMI T0	BMI T12		Sniffin sticks T0	Sniffin sticks T12
*Median*	26.00	26.00	*Median*	5	5
*IQR*	3.32	4.46	IQR	7.75	9
	BMI T12-T0			Sniffin sticks T12-T0	
*Comparison (p)* ^*e*^	0.107		*Comparison (p)* ^*e*^	0.532	
**Smell improvement group** ^**d**^ **(** ***n*** ** = 72)**					
	BMI T0	BMI T12		Sniffin sticks T0	Sniffin sticks T12
*Mean*	25.34	25.73	*Median*	3.76	11.51
*SD*	3.97	3.87	*IQR*	3	3
	BMI T12-T0			Sniffin sticks T12-T0	
*Comparison (p)* ^*f*^	< 0.001		*Comparison (p)* ^*e*^	< 0.001	

^a^ Anosmic group intended as score of 0–5/16 at T12 evaluation. ^b^ Hyposmic group intended as score of 6–11/16 at Sniffin’ sticks test at T12 evaluation. ^c^ Olfactory-stable group intended as patients whose olfaction decreases, stays stable or does not improve of more than 2 points at T12 Sniffin stick test evaluation. ^d^ Smell improvement intended as improvement of more than 2 points at T12 Sniffin stick test evaluation. ^e^ Analysis was made using Wilcoxon signed-rank Test since the non-gaussian distribution of the variables. ^f^ Analysis was performed using T-paired Test since the gaussian distribution of the variables

## Discussion

Dupilumab appears to be the most effective biologic treatment for CRSwNP regarding the improvement of smell, with significant increases in subjective and semi-objective scores already observed at the 1-month follow-up [22–24]. As expected, considering real-life data on Dupilumab, we found a significant improvement in olfactory function in our case series (*p* < .001). The physiopathological mechanisms underlying olfactory impairment in CRSwNP patients and the mechanisms underlying olfactory recovery after therapy with Dupilumab are in part unrelated to the volume and flow-halting effect of the polyps and might be mainly correlated to the resolution of local inflammation. Some authors assert that olfactory sensory neurons (OSNs) are particularly susceptible to local immune mediators in the setting of CRSwNP, like IL-4, IL-5, IL-13, and eosinophils [25, 26]. Infiltrating immune cells in the olfactory neuroepithelium might account for the dysfunction of the peripheral olfactory system [26]. This might explain how the amelioration in olfaction could be led both by an improvement of conduction towards the olfactory epithelium and by the decrease of neuroinflammation.

Odors in the surrounding environment significantly influence an individual’s capacity to recognize and identify food sources, subsequently eliciting various appetite responses [27]. A recent review by Zhang et al. highlighted how the characteristics of the olfactory stimuli (e.g., the congruency between the olfactory perception and the foods, intensity and duration of exposure to smells, and taste properties of odors) modulate the effects on food behavior [28]. Consequently, overweight people show a heightened response to appetite and hence food intake when explicitly exposed to food smells compared to normal-weight patients [27, 29, 30].

Nevertheless, in our series, all patients started with a normal BMI or were overweight but not obese at baseline, and none of them encountered so much gain as to change their weight status (e.g., from normal-weight to overweight/to obese). Therefore, even if the olfaction restoration may have significantly influenced weight variation, it appears not to be clinically significant during our follow-up period. Even though weight gain is not a known or described side effect of Dupilumab treatment, there are some preliminary reports that explore this possibility in atopic dermatitis [31]. The aim of our study is therefore not to link the treatment itself to weight gain but the restoration of olfaction to an increase in appetite and therefore a secondary weight gain.

We found a significant increase in our case series’ BMI (p = .002) and, given the unavailability of real-life comparisons, we tried to further investigate our findings. We divided our case series in terms of smell variation to see if there is a link to weight gain. Among patients who remained anosmic after 1 year of treatment (n = 13, 13.5%), there was no statistically significant change in BMI values (p = .201). Also, patients whose olfaction decreased, stayed stable, or did not improve by more than 2 points at the Sniffin’ Sticks test did not have a significant increase in their BMI score (*p* = .107). Of note, that also these patients that are non-responders to the treatment regarding olfaction had significant improvement of SNOT (*p* < .001) and NPS (*p* < .001) scores, showing that these parameters are independent of each other.

Accordingly, when considering only patients who had a satisfactory improvement in smell (*n* = 72, 75%), these presented with a statistically significant increase in their BMI index (*p* < .001). Patients who ended up at T12 with hyposmia or normosmia reported an overall improvement in smell (*p* < .001 both) and accordingly an increase in BMI (*p* < .001 both).

The relationship with food is complex and a combination of many factors has to be taken into account. Certainly, the cause of weight gain should not be reduced only to an improvement in the sense of smell, even though it may be in this specific scenario the leading cause. The emotional sphere, physical activity, genetic predisposition, and other factors interact to influence weight. Indeed, we have to consider the secondary effects of improved health due to Dupilumab: there certainly is evidence of better nasal airflow, which could lead to an increase in physical activity and fitness, and hence to weight loss [32]. Furthermore, none of our patients used oral corticosteroids (OCS) during the follow-up period, which are known to increase the risk of weight gain: we initially expected to observe a weight loss in this case series, which eventually did not occur. Also, in the current literature, the association of OCS dose and duration with increased weight risk is not well-quantified [33].

This shows the many contrasting factors at play in this field: further studies could shed light on how these drivers play a role in weight maintenance and fluctuation. A potential bias of our study is the lack of a control group to observe the natural progression of the disease and its consequences on BMI.

Furthermore, we did not consider other potential influencing factors in the weight variation, such as any change in physical activity or any other external factors in daily life that may interfere with patients’ metabolism. Also, smoking was not analyzed statistically as we only had very few smokers in our cohort.

## Conclusions

This study found a significant increase in BMI among patients with olfactory restoration under Dupilumab treatment for CRSwNP, prompting an exploration of the relationship between olfactory improvement and weight gain. While the study suggests a correlation between smell improvement and weight gain, the complex nature of weight regulation, influenced by factors such as emotional well-being, physical activity, and genetics, is acknowledged. It emphasizes the need for further research to delve into the molecular pathways involved, the specific causal relationship, and long-term implications.
